# Comparative Analysis of Volatile Organic Compound Purification Techniques in Complex Cooking Emissions: Adsorption, Photocatalysis and Combined Systems

**DOI:** 10.3390/molecules28227658

**Published:** 2023-11-18

**Authors:** Daniele Zatta, Mattia Segata, Franco Biasioli, Ottaviano Allegretti, Giovanna Bochicchio, Roberto Verucchi, Francesco Chiavarini, Luca Cappellin

**Affiliations:** 1 Department of Chemical Sciences, University of Padua, Via Marzolo 1, 35131 Padua, Italy; daniele.zatta.1@studenti.unipd.it; 2 3S Lab S.r.l., Via dei Zeni 30, 38010 Cavedago, Italy; mattia.segata@3slab.eu; 3 Department of Food Quality and Nutrition, Research and Innovation Centre, Edmund Mach Foundation, Via Mach 1, 38010 San Michele all‘Adige, Italy; franco.biasioli@fmach.it; 4 Institute of BioEconomy, National Research Council (CNR-IBE), Via Biasi 75, 38010 San Michele all’Adige, Italy; ottaviano.allegretti@ibe.cnr.it (O.A.); giovanna.bochicchio@ibe.cnr.it (G.B.); 5 Institute of Materials for Electronics and Magnetism, National Research Council (CNR-IMEM), Via alla Cascata 56/C, 38123 Povo, Italy; roberto.verucchi@imem.cnr.it; 6 Elica SpA, Via E. Casoli 2, 60044 Fabriano, Italy; f.chiavarini@elica.com

**Keywords:** VOCs, PTR-MS, adsorption, photocatalysis, cooking emissions

## Abstract

Volatile organic compounds (VOCs) are molecules present in our everyday life, and they can be positive, such as in the formation of odour and food flavour, or harmful to the environment and humans, and research is focusing on limiting their emissions. Various methods have been used to achieve this purpose. Firstly, we review three main degradation methods: activated carbon, photocatalysis and a synergetic system. We provide a general overview of the operative conditions and report the possibility of VOC abatement during cooking. Within the literature, none of these systems has ever been tested in the presence of complex matrices, such as during cooking processes. The aim of this study is to compare the three methods in order to understand the behaviour of filter systems in the case of realistically complex gas mixtures. Proton transfer reaction–mass spectrometry (PTR-MS) has been used in the real-time monitoring of volatilome. Due to the fact that VOC emissions are highly dependent on the composition of the food cooked, we evaluated the degradation capacity of the three systems for different burger types (meat, greens, and fish). We demonstrate the pros and cons of photocatalysis and adsorption and how a combined approach can mitigate the drawbacks of photocatalysis.

## 1. Introduction

During cooking, many volatile organic compounds (VOCs), chemicals that easily evaporate at room temperature, are continuously produced and released into the air. Some of these molecules are fundamental in food characterization because they contribute to both flavour and odour formation [1]. On the one hand, VOCs play a vital role in the aroma and taste perception of food products, contributing to their overall sensory quality; however, on the other hand, some of these compounds are known human carcinogens, and other suspected carcinogens are also under study. For this reason, research is focused on reducing harmful VOC emissions, using various methods to reach this goal.

The production of VOCs in food is a complex process influenced by various factors, such as food composition, processing conditions, and microbial activity. The primary process regarding food cooking is the Maillard reaction: a complex chemical reaction that occurs between amino acids and reducing sugars, usually at elevated temperatures. The reactants condensate to form a glycosylamine, which rearranges to form an Amadori compound if the sugar is aldose or a Heyns compound in the case of ketose. Then, these compounds react with other molecules, such as amines and hydrogen sulfide, to produce various VOCs compounding food flavour and odour [2]. Strecker degradation is relevant in the subset of final Maillard reactions. In fact, α-amminoacid can react with dicarbonyl molecules to form aminoketone, which acts as a precursor of various heterocyclic compounds, such as thiophene, pyrrole, and furan [3]. The last major VOC production mechanism during cooking is lipid oxidation, a radical reaction catalysed via an enzymatic or non-enzymatic pathway in the case of autoxidation, regarding mainly unsaturated fatty acids, which decompose to stable end-products, such as aldehydes, ketones, and alcohols. A common example of this reaction is the oxidation of linoleic acid, which, thanks to singlet oxygen, generates hydroperoxide. This intermediate can undergo cleavage to obtain aldehydes, such as hexanal [4].

Maillard reaction and lipid oxidation by-products can react together, leading to new and complex heterocyclic molecules. Henderson and Nawar noticed that the reaction between 2–4 decadienal, produced via the cleavage of 9-hydroperoxide of linoleic acid and Maillard intermediates of valine, produces 2-pentylpyridine [5]. Volatiles released by this cross-reaction contribute slightly to odour due to their weak odour intensity and high perception threshold.

It is already known that cooking processes produce several types of VOCs, among which several can be of concern in terms of the environment and long-term health, such as formaldehyde, methanol, acetaldehyde, acetone, and acetic acid [1,6]. Two typical methods of VOC degradation for indoor applications are activated carbons (AC)s and photocatalysis (PC). However, these two methods have some drawbacks, such as high energy consumption and environmental incompatibility [7].

Various works have already demonstrated the possibility of reducing the amount of VOCs using activated charcoal. Adsorption is the phenomena of gases or solutes being absorbed onto the surface of a solid or liquid support. Adsorbents come in a wide variety of forms, including zeolite, polymeric resins, alumina, and silica structures. Activated carbon, produced via carbonization and activation, is the most used adsorbent material because of its great surface area and chemical stability, especially towards acids and alkalis. Because of their hardness and stability, nutshells, such as those from coconut and walnut are frequently employed as precursors. Additionally, it has a natural porosity that makes activation simple [8]. Then, to improve surface area and pore volume, the coal undergoes a process of physical or chemical activation. In fact, usually, a greater surface area leads to enhanced adsorption capacity. Gases at fixed temperatures and pressures, such as water vapour or CO_2_, are widely used as activating agents. Thus, AC possesses improved VOC adsorption capacity, greater surface area, specific pore size, and surface chemical functional groups [9]. Regarding pore volume, the most effective adsorption occurs when the pore diameter is a little larger than the diameter of VOCs. In fact, the adsorption force between AC and the pollutant is weak when the pore is far larger. VOCs usually have a diameter similar to the micropore scale, and, consequently, the number of micropores is a key factor in terms of VOC adsorption, especially at low concentrations [10]. Mesopores are important as well due to their greater diffusion capacity towards VOCs than micropores. The mechanism of adsorption over activated carbons involves a charge transfer from VOCs to coal, which leads to electrostatic attraction. Then, interactions between polar VOCs and hydrophilic sites occur, as well as among nonpolar VOCs and hydrophobic sites. At the same time, a partition equilibrium is established between VOCs and the non-carbonized char phase. For example, methanol and acetone are adsorbed by polar groups on the surface thanks to dipole–dipole interaction. Thus, AC shows both adsorption and partition effects in air cleaning [10,11]. There are many limitations on the use of AC as an adsorbent; firstly, if the adsorption occurs at elevated temperatures, AC can ignite, or its porous structure can collapse [8]. Secondly, when humidity is above 50%, competitive water adsorption will form a layer, thus making the surface become hydrophilic, switching the class of catchable VOCs. Furthermore, trapped water can displace adsorbed VOCs and react with them or form a two-phase solution with a partition equilibrium. Chemisorption and irreversible sorption can also occur, especially at high VOC concentrations and high moisture. All previously mentioned issues may, in the worst-case scenario, result in reduced adsorption capacity [10,12,13].

Recently, intense research on improving the metal oxide bulk or nanoparticles as photocatalysts has been carried out. All of the investigated compounds have semiconductor metal properties, such as a particular electronic structure, flexibility, high photocatalytic activity, and adsorption capacity. They also show great chemical stability against acids and alkalis [14]. The process involves four steps: (1) UV sources or visible light causes electronic promotion, forming an electron–hole pair (e^−^/h^+^); (2) the adsorption of VOC compounds; (3) Redox reactions charged to H_2_O and O_2_, leading to the release of reactive oxygen species (ROS), such as hydroxyl radicals (∙OH) and superoxide radical anion (∙O_2_^−^), both strong oxidants; (4) the degradation of VOCs due to ROS oxidation, which converts organic compounds into carbon dioxide and water in several steps [15]. PC can occur at room temperature, but it oxidizes low concentrations of volatile compounds and has low durability due to coke fouling. For this reason, thermocatalysis is recommended to avoid catalyst poisoning and improve degradation rate [16]. Between these materials, TiO_2_ shows nontoxicity, high chemical and thermal stability, strong oxidizing power, best biocompatibility, and greater catalytic activity in relation to defects [17,18]. Titania is a semiconductor with a band gap of 3.2 eV, corresponding to the wavelength of 390 nm, which requires UV irradiation to achieve electron excitation. The broad energy gap and recombination between electron–hole pairs are a drawback of photocatalytic materials. The most common method to avoid these problems is doping the semiconductor with noble metals, such as Ag and Au, and because of this, titania exhibits a strict band gap and an increased exciton lifetime [19]. A major limitation to PC is the formation of reaction intermediates. In fact, some oxidation steps of complex VOCs can lead to unwanted intermediates, such as the most common ones: formaldehyde, acetone, benzaldehyde, ethanol and benzyl alcohol, or other toxic molecules. Particularly when used in situations where there are large quantities of VOCs, these by-products can saturate the active sites of the catalyst, which leads to catalyst deactivation and poisons the user.

A possible way to avoid both drawbacks of the aforementioned methods is to combine adsorption and photocatalysis in carbon-based nanocomposites, such as activated carbon coupled with nano-TiO_2_ (TiO_2_/AC) or activated carbon fibres (TiO_2_/ACF). Due to the lack of polar surface functional groups in AC and ACF, only nonpolar and weakly polar substances, such as toluene and formaldehyde, could be removed. A possible advantage of carbon-based nanocomposite paired with photocatalysis is the prevention of the generation of intermediates, which are immediately captured by charcoal, and the inactivation of the catalyst. In addition, due to its high adsorption potential and fast charge transfer, activated carbon holds VOC molecules in the proximity of the active sites, and it promotes the generation of radical ROS [7]. It would also be conceivable to modify the carbon surface to bring about chemical alterations that increase the interaction between VOCs and the ACF surface. On the other hand, AC-based nanocomposites have the benefit of in situ regeneration, but it is highly dependent on the size of the micropores, which, when left unaltered, reach dimensions of 2 nm, making it impossible for larger molecules to be adsorbed and leading to the failure of the AC synergy/PC. By modifying the material through acid treatment or water vapour gasification, the ratio of mesopore to micropore can be increased, and as a result, the treated material also exhibits improved mechanical strength [20]. As cited above, the separation between holes and electrons is crucial for system operativity. Temperature is another key variable in adsorption–photocatalysis. Heterogeneous adsorption is an exothermic process; therefore, a low temperature helps VOC sorption. Diffusion, instead, is endothermic and, therefore, decreasing temperature hinders the diffusion of the adsorbed compounds in the internal porosity.

In conclusion, an interesting challenge, which, to the best of our knowledge, has not been addressed within the literature, is the evaluation of the behaviour of AC/PC technology related to a complex mixture of VOCs, focusing on the emissions generated during cooking. In such a scenario, it would be worth investigating the compensation of the defects present in each method when used alone and if VOCs with low adsorption rate in AC are oxidized by PC. Furthermore, it would be important to evaluate the intermediate compounds and their possible interference with air cleaning processes.

The gold standard of VOC analysis is gas chromatography coupled to mass spectrometry, which provides remarkable compound separation and identification but it is slow and time-consuming. On the contrary, proton transfer reaction–mass spectrometry (PTR-MS) enables VOC detection with higher time resolution in the order of tens of seconds/minutes for quadrupole mass analysers and a split second for time-of-flight mass analysers. It is based on the chemical ionization of VOCs through the use of primary ions, typically H_3_O^+^, which have the peculiar property of not reacting with the major components of air (N_2_ and O_2_) but can react with VOCs with a higher proton affinity than water. PTR-MS exhibits great sensitivity, with detection limits reaching parts per trillion by volume (ppbv) and enables real-time VOC monitoring. On the other hand, PTR-MS separates protonated VOCs only on the basis of their *m*/*z* values, leading to the difficult qualification of the compounds. In comparison, GC-MS enables a better identification and quantification of complex mixtures due to chromatographic resolution. However, it is time-consuming due to the fact that the typical analysis time is about one hour per sample. Another advantage of PTR-MS is the absence of sample pre-treatment and analyte preconcentration steps, contrary to GC-MS. VOC monitoring with GC systems typically needs sample trapping [12]. Then, a desorption procedure must be applied to inject the analytes into a GC-MS system. Two common techniques are solvent desorption and thermal desorption. Thermal desorption needs both preconcentration of VOCs, usually carried out with cryofocusing, and maintenance of the cold chain until analysis [21]. A major problem of this method is the presence of water in a matrix, which can be transferred into a column, leading to serious problems and, therefore, must be removed. For these reasons, GC-MS requires several pre-treatment operations, and the analysis must be performed with particular caution.

This work aims to compare the performance between adsorption, photocatalysis, and combined systems in abating the complex VOC matrices produced when cooking three different types of hamburgers: meat, greens, and fish. The objective of this research is to determine whether the synergy between adsorption and photocatalysis is useful in the removal of VOCs from indoor air for residential usage. Climatic chambers were used to imitate domestic kitchens. The air purification system that was employed for this project was created in view of possible applications in fume hoods in the future. PTR-MS was employed to monitor VOCs in real-time, thus providing time-resolved data on VOC emissions and abatement. Several studies [1,22,23,24,25] have already demonstrated the possibility of PTR-MS on VOC monitoring and quantification, and in this work, we also aim to show the potential of this technique to evaluate the performance of air cleaning systems in the abatement of complex VOC mixtures, such as the ones produced upon cooking.

Indoor air remediation has been previously investigated in several studies; however, to the best of our knowledge, none of these have ever been monitored with PTR-MS during cooking processes [10,26,27,28].

## 2. Results and Discussion

### 2.1. Principal Component Analysis

**Figure 1:**
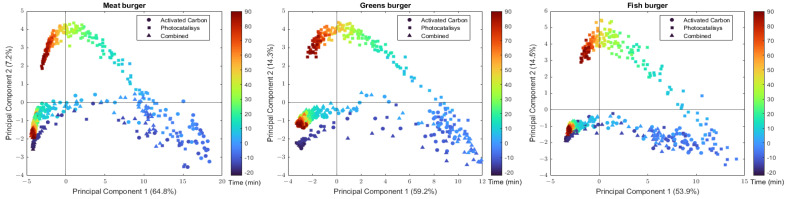
PCA score plot PC2 vs. PC1. The three abatement systems are reported in the same plot. The blue colour refers to the start of measurement, before powering on the cleaning system, while the red colour refers to the end measurement.

To offer a comprehensive view of the global dataset, we conducted separate PCAs for each type of burger, representing all three abatement systems together on a single plot (Figure 1). The scores show interesting trends: upon cooking, the VOCs in the chamber change dramatically; however, when the purifying system is turned on, the VOC composition in the chamber either almost returns to the starting point (in the case of “Activated Carbon” and “Combined”) or moves away (in the case of “Photocatalysis”). It is worth noticing that in no case did the original VOC configuration seem completely re-established after 90 min of purifying system operation. This is particularly evident in the case of vegetal burgers (Figure 1, central panel). Regardless, the synergetic setup seems to be able to compensate for the negative aspects of photocatalysis. In fact, the results of “Activated Carbon” and “Combined” are rather similar.

### 2.2. Time Trends

The trend of the five compounds that showed the highest concentration was specifically reported as well as the “Total VOC” concentration, which was measured as the sum of all spectral signals. The selected compounds were acetic acid (protonated ion signal at *m*/*z* = 61), acetaldehyde (*m*/*z* = 45), formaldehyde (*m*/*z* = 31), methanol (*m*/*z* = 33), and acetone (*m*/*z* = 59). *m*/*z* = 43 has been discarded since it is a non-specific fragment and, therefore, difficult to attribute to a single compound. Evidence of these VOCs being detected through the use of PTR-MS was reported by Cappellin et al. with regard to methanol, acetaldehyde and acetone [23], and by Ni et al. with regard to acetic acid and formaldehyde [1]. The simultaneous measurement of formaldehyde, acetaldehyde, and methanol was carried out by Stucchi et al., who demonstrated not only the possibility of multi-VOC monitoring but also that photocatalysis on titania powder is suitable for volatile degradation [25]. This work is also interesting because they studied the photodegradation of 17 different VOCs, which is similar to the complex gaseous matrix we want to investigate.

A first comparison of concentration vs. time plot can be carried out in relation to emissions: until time = 0, the abatement system is turned off to achieve air homogeneity. Confirmation of data repeatability is given by the similar trend for each type of burger despite the method used. Instead, it can be noticed that cooking different types of patties leads to great differences in terms of the initial VOC profile, probably because of the different compositions of the burgers, such as their carbohydrates and fatty acids.

#### 2.2.1. Formaldehyde

**Figure 2:**
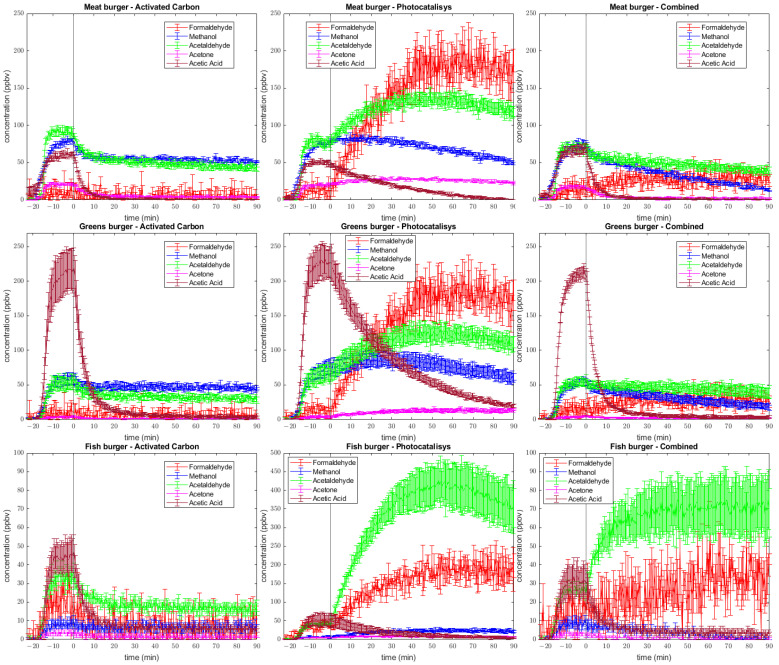
Concentration vs. time plot of five selected VOCs. There are reported the three types of burgers and for each, the three purifying methods. Data are means ± standard error (*n* = 3).

In all types of burgers, formaldehyde (*m*/*z* = 31, indicated by the red line) is produced at a low concentration, stabilizing at a few ppb before the purification system is turned on (Figure 2). Over time, when using activated carbons as adsorbents, the amount remains almost constant. Conversely, when operating with photocatalysis, formaldehyde increases to 200 ppb in all three burger types, presumably due to its formation as an oxidation reaction intermediate, such as in methanol oxidation [29]. In the combined system, however, the concentration trend returns to being almost a plateau at ca. 25 ppbv, indicating great compensation from activated carbon. ACGIH suggest threshold limit values as being time-weighted over 8 h (TLV-TWA), short time exposure (TLV-STEL), or a ceiling limit (TLV-C). For formaldehyde, which is a confirmed human carcinogen, they report TLV-STEL of 300 ppb and TWA of 100 ppb. Therefore, the use of a photocatalytic oxidation filter can configure a health risk due to the significant production during this time. In “Activated Carbon” and in “Combined”, even if there is a stabilization at higher values, both show values lower than the suggested ACGIH values. Thus, indicatively, the risk is low when using this filter as the purifying system.

#### 2.2.2. Methanol

Methanol (*m*/*z* = 33) is detected before the activation of the purification system in different amounts depending on burger composition since it is readily produced upon cooking. The highest production is found in the case of the meat burger (around 80 ppbv) followed by the vegetable burger (50 ppbv) and fish burger (10 ppbv).

In meat burgers, we report that activated carbon reaches site saturation in a short time. As expected, methanol concentration does not decrease upon the activation of the activated carbon filter. Despite the low abatement rate, the photocatalysis approach has a better profile and seems partially capable of degrading this VOC after an initial plateau. The combined system has the best trend and shows continuous decrease after being switched on, with a higher degradation rate. Adsorption on activated carbon in vegetal burgers keeps the methanol amount constant at 50 ppb. In all cases of “Activated Carbon” and “Photocatalysis”, at the end of 90 min of air purifier activation, methanol concentration is still high, while the “Combined” method is more effective in the case of the fish burger for which Figure 2 displays complete air purification from methanol in 90 min. In the case of the meat burger, which produced a higher methanol concentration upon cooking, the “Combined” method is still the most effective but would require longer than 90 min for a complete air cleansing. Methanol is not classifiable as a human carcinogen; ACGIH suggests TLV-TWA of 200,000 ppb, which is far away from our values (90 ppb ca. peak). Thus, we can assume a low-risk exposure.

#### 2.2.3. Acetaldehyde

Acetaldehyde (*m*/*z* = 45) has the most varied behaviour among the considered VOCs. It is produced upon cooking meat burgers (ca. 80 ppbv), vegetable burgers (50 ppbv), and fish burgers (30 ppbv). A possible explanation of this behaviour could be the presence of reactions that occur during the cooking process, such as Maillard and Strecker degradation of proteins. The Maillard reaction is a reaction that happens during cooking and involves a free ammino group of an amino acid and a carbonyl one of carbohydrate. The final products of this process are various classes of molecules compounding the aliment flavour. Another crucial step in flavour generation is Strecker degradation, which is also a crucial step in Maillard’s reaction. α-amminoacid reacts with a dicarbonyl molecule to form an aminoketone, which acts as a precursor of heterocyclic compounds responsible for food flavour and odour [2]. The principal end-product of Strecker degradation is bonded aldehyde and amminoacid, which are known to lead to acetaldehyde production and are mainly glycine and alanine. Previous works [30,31] have highlighted how acetaldehyde can be released by foods that contain high protein and fatty acids with a high degree of unsaturation and a high content of monosaccharides and disaccharides, such as fructose and sucrose.

A very different behaviour is observed between the outcome of the air cleaning by the three devices on the three different types of burgers.

The first anomaly that can be noticed compared to the other volatiles is in the photocatalysis technique applied to the fish burger: after the conditioning of the sampling space with the cooked burger volatilome and the ignition of TiO_2_ UVA LED, a significant production of acetaldehyde occurred, with a peak of almost 450 ppbv. These are the highest concentration values detected during the experiments. Therefore, the “Photocatalysis” air cleaning system produced a high amount of acetaldehyde as a by-product, which pollutes the chamber.

In the activated carbon filter applied to the cooking emissions of the fish burger, the acetaldehyde amount is small and constant during time, likely due to the saturation of coal porosity. As it emerges from the combination with photocatalysis, the introduction of carbon as an adsorbent material enables limiting the negative effects of photodegradation, keeping its value stable over time. In the experiments using the meat burger, upon the activation of the air cleaning system, acetaldehyde follows the trend of those measured for the fish burger, but with smaller concentration values, which is why adsorption with coal in combined system mitigates the influence of photocatalysis. In the case of the vegetable burger, no increase in acetaldehyde is detected when using the “Combined” system, while, as expected, there is a small increase when using the “Photocatalysis” system.

Interferences with the acetaldehyde signal from other molecules are expected to be minor. CO₂ could have an interfering signal, which could lead to the overestimation of the amount of acetaldehyde. According to Cappellin et al. (2019), since CO₂ does not react at a collision rate with H_3_O^+^, it is ionized very inefficiently compared to acetaldehyde. In fact, the sensibility toward CO₂ is about 0.0001 cps/ppbv, while for acetaldehyde, it is 10 cps/ppbv [32]. Therefore, even if all VOCs (<1 ppmv) were converted to CO₂ via photodegradation, the maximum values of cps would be <0.1, corresponding to interference of <0.01 ppbv on the acetaldehyde signal. This value, compared to the ca. 450 ppbv of the detected acetaldehyde, would be negligible. Another contribution to *m*/*z* = 45 could come from the ionization of butyraldehyde (*m*/*z* = 73) for the loss of the carbonyl group (*m*/*z* = 28) in the ionization and fragmentation process [33], but the phenomenon is appreciable only in the case of fish burgers because in the others butyraldehyde is not detected over the background. In addition, it causes a negligible effect since *m*/*z* = 73 is minor compared to *m*/*z* = 45, and the expected fragmentation is 1:1. A similar argument on complex aldehyde, in general, allows us to assume that these compounds are negligible in acetaldehyde quantification. Acetaldehyde is reported by ACGIH as a suspected human carcinogen, and for this reason, they assume it to be TLV-C 25,000 ppb. Then, our most intense value is ca. 450 ppb, indicating a low-risk situation despite the significant production of this VOC released via photocatalytic oxidation.

#### 2.2.4. Acetone

It can be noticed that acetone (*m*/*z* = 59) is present in almost every sample around the baseline, except for the meat measurement, where there is a little production of this VOC upon cooking. The “Photocatalysis” method does not decrease its concentration. However, similarly to previous cases, coupling with activated coal restores its values around a few ppb. ACGIH, regard its classification as “not classifiable as Human Carcinogen”, suggest a TLV of 250,000 ppb (TWA) and 500,000 ppb (STEL) for acetone. Therefore, with the highest value of 25 ppb, exposure risk seems to be negligible.

#### 2.2.5. Acetic Acid

Acetic acid (*m*/*z* = 61) is another of the most intense VOCs generated upon cooking, and a possible source of its production, as with the other compounds, can be traced back to the Maillard reaction [34]. Another source could be the oxidation of acetaldehyde, although its contribution to the overall quantity is relatively minor. The greens burger produces the highest ethanoic acid amount upon cooking, but on par with the other burgers, after switching on of the purifying system, the concentration moves quickly towards zero. The “Combined” system has a higher slope compared to the separate techniques. Thus, synergetic coupling seems to be more efficient for acetic acid. Comparison with TLV (15,000 ppb in STEL and 10,000 ppb TWA) suggests that even in greens burgers, which show a peak value of ca. 250 ppb, it has a low risk in terms of health.

#### 2.2.6. Clean Air Delivery Rate

In order to offer a quantitative insight on the efficiency of the three indoor air cleaning systems, we calculated the Clean Air Delivery Rate (CADR) as follows:

(1) $$\;CADR=\left(k_{t}-k_{n}\right)\;V\;\left[m^{3}h^{-1}\right]$$

*V* is the volume of the chamber (8 m^3^), *k_t_* is the pollutant decay rate while the air cleaning device is operating (h^−1^), and *k_n_* is the natural pollutant decay rate in the test chamber [35]. *k_n_* is approximated as being 0 since the chamber losses were negligible, while *k_t_* has been calculated using an exponential decay from 0 to 12 min, using the following equation.

(2) $$c_{t}=c_{0}e^{{tk}_{t}}\;\left[cps\;corr.\right]$$

where *c_t_* is the pollutant concentration at a certain time, and *c*_0_ is the average amount of the first seven values.

**Figure 3:**
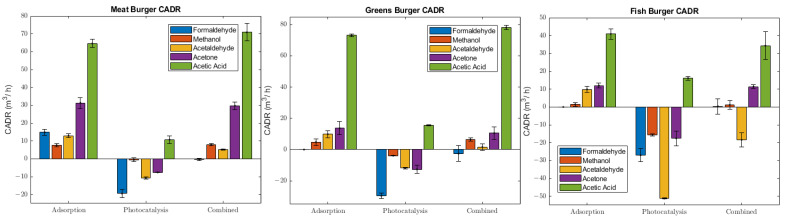
CADR bar plot for each type of burger and sorted by technique.

The negative CADR values suggest that photocatalytic oxidation is inefficient and generates some VOCs. This is particularly highlighted in the fish burger for acetaldehyde (Figure 3, right panel), corresponding to the high increase rate, which led to a 450 ppbv peak. Acetic acid, in both “Adsorption” and “Combined” techniques, displayed significant values in terms of CADR, corresponding to an elevated decrease rate in the first minutes of purification system activity. In photocatalysis, instead, its value is still positive but lower than the other techniques. Formaldehyde CADR calculation in the adsorption case for greens and fish burgers is not applicable due to the low values of this pollutant. In the case of the “Combined” system applied to the fish burger, formaldehyde shows a slight positive CADR, while acetaldehyde CADR is negative but higher than that for “Photocatalysis”.

A limit of CADR scores is the limited time span of their calculation. In fact, 12 min could be nonrepresentative of the situation during system operation.

### 2.3. Total Volatile Organic Compounds (TVOCs)

**Figure 4:**
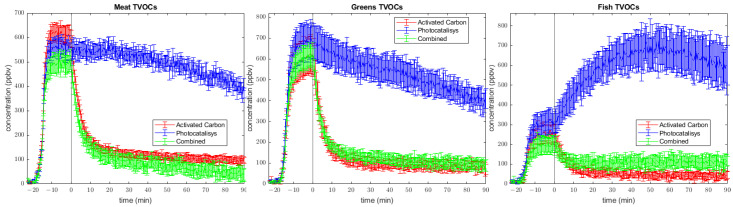
Total VOC profile of three burger types during cooking (time < 0) and after the activation of air purification system (time > 0).

Some other conclusions can be elaborated on the total VOC (TVOC) plot (Figure 4). First of all, they can be used to confirm the initial statement based on the PCA plots. In fact, all three burger types show similar trends. After switching on the abatement system, the Activated Carbon and Combined systems have similar TVOC values for most of the sampling times, except for the meat burger, for which the synergy between AC and PC is better than that when using only coal. For both meat and greens, using the photocatalysis method, a reduction in the total amount of VOCs over time is displayed, although the decrease rate is lower than for the other two techniques. On the contrary, the same procedure with fish patties leads to a significant increase in volatile concentration, mainly dependent on acetaldehyde production via photocatalysis. Looking at volatile production during the cooking phase, it can be noticed that there are different values of TVOC concentrations. This can be the effect of the different chemical compositions of patties rich in carbohydrates, proteins, and lipids. These plots suggest that synergetic coupling yields better results in the meat burger, leading to TVOC concentration at 90 min under the values obtained from activated carbon measurements; meanwhile, for greens burgers, there is not a significant difference. Regarding fish patties, photocatalysis exhibits lower filtering performance due to the high emissions of intermediates (formaldehyde and acetaldehyde, Figure 2), meaning that the combined setup is slightly less effective than pure activated carbon alone due to the stabilization of formaldehyde and acetaldehyde values at ca. 70 ppbv and 25 ppb.

## 3. Materials and Methods

All filters used as air purifier were manufactured by Elica S.p.A (Fabriano, Italy). The photocatalytic system is composed of 2 tiles of titan dioxide 55 × 55 × 10 mm, with 4 LED UVA each (peak at 367 nm) and powered using 3.6 V. It has a TiO_2_ loading weight of 2.5 ± 0.5 g, an LED power of 20.8 W, and an average irradiance of 28 mW cm^−2^. The manufacturer also reports a Radiant Flux of 1.0 W.

The adsorption material is a ceramic-reinforced activated carbon composite; a single filter is composed of 4 honeycombs 48 × 48 × 40 mm, with a cell density of 676. The manufacturer reports a low pressure drop and high regeneration in an oven (200 °C, 45 min)

Measurements were performed in an 8 m^3^ polyethylene (PE) chamber, and, before the cooking experiments, ambient sampling was conditioned with purified air. The pollutant abatement systems (activated carbon, photocatalysis, or both) were installed in the middle of the chamber beside a hotplate. The meat hamburgers used to monitor the VOC emissions were made of adult bovine (produced by CEM SOC.COOP Cesena, Italy). The greens burgers in use are “Fior dì natura^®^” (Eurospin, San Michele all’Adige, Italy.). Lastly, fish burgers based on rainbow trout (Astro, San Michele all’Adige, Italy) were purchased from a local supermarket. In each measurement, after conditioning the chamber, a burger was placed in a pan and cooked on a hot plate with a set power of 1000 W for 5 min. The plate was then switched off, the air in the chamber was given 15 min to homogenise, and then the purification system was turned on for 90 min. This value of the sampling time was chosen because it is comparable with the median working time of an extractor hood for home use during cooking. Each step could be triggered remotely by an operator so that it was not necessary to enter the chamber during the whole experiment.

The instrument used for VOC measurement is a PTR-MS (Ionicon Analytik GmbH, Innsbruck, Austria) equipped with a quadrupole detector directly linked to the chamber via Teflon PTFE tubing (1/8”). The sampling flow was set to 40 sccm. The method used to collect the data involves a full scan from 20 *m*/*z* to 250 *m*/*z* and a scanning time of 200 ms for each mass. For every combination of hamburger type–purification system, we performed three replicates. Every measurement was run in compliance with the rules on the measurement of the efficiency of photocatalytic devices used for the elimination of VOCs in indoor environments [36] and on performance measure of air cleaners [37]. Calibration curves with a pure standard injected into the chamber were constructed for the selected VOCs (acetaldehyde, formaldehyde, and acetone), while for the other compounds, reaction kinetics were used to predict the response factors explained by Cappellin et al. [23]

The instrumental response of PTR-MS is measured in [cps or counts per second], which has then been translated into concentration (ppbv or parts per billion by volume) using calibration lines. For each replicate, we subtracted the background, computing this using the mean of the first seven time points of each measurement. Then, for each time point, the mean of the replicate signals was calculated, and its associated uncertainty was evaluated as the standard error of the mean. In-house routines written in MATLAB^®^ (R2023a) were employed to perform Principal Component Analysis (PCA) for data exploration. Before applying the calculation function of PCA, the dataset of the three methods, already averaged, was centred and normalized against standard deviation. Every column with a null standard deviation, corresponding to masses that cannot be detected with PTR-MS, has been considered irrelevant and, therefore, excluded from the PCA.

## 4. Conclusions

We successfully applied Direct Injection Mass Spectrometry (DI-MS) for the real-time assessment of the effectiveness of major indoor air treatment methods. Particularly, PTR-MS appears to be a promising technology for volatile organic compound (VOC) monitoring, even in challenging scenarios, such as cooking emissions from complex matrices. We conducted analyses on three complex matrices representing significant food categories or relevant situations in homemade cooking. Our findings indicate that formaldehyde levels generated by photocatalysis lead to a critical situation. On the contrary, activated carbons and a combined system led to lower levels of this pollutant, posing a low risk according to the TLV values suggested by ACGIH. Acetaldehyde exhibits similar trends to formaldehyde. The trend of methanol suggests differences between the three methods: in activated carbons, possibly due to saturation, its value remains constant, while the hybrid system slowly degrades methanol. Photocatalysis exhibits a distinct behaviour, with the concentration initially increasing slightly and then decreasing over time, indicating poor performance. In contrast, acetic acid is effectively degraded by all of the tested systems.

Referring to the Threshold Limit Values-Time-Weighted Average (TLV-TWA) for an 8 h period, the Short-Term Exposure Limit (TLV-STEL) for a 15 min interval, or TLV-C (maximum) defined by ACGIH as acceptable exposure levels for workers to chemicals without adverse effects, the five selected VOCs consistently exhibited values below the specified thresholds in all measurements, except for the photocatalysis system in formaldehyde cleansing. In conclusion, the combination of activated carbons and photocatalysis does not improve the air cleaning performance compared to activated carbons for the studied VOCs, except for methanol, which is more effectively removed over 90 min. On the contrary, the combined system is more effective than using photocatalysis alone and the drawbacks of photocatalysis in terms of the by-products of VOC generation are mitigated but not completely removed.

## Data Availability

The data presented in this study are openly available in Research Data UniPD at www.doi.org/10.25430/researchdata.cab.unipd.it.00001058.
